# AMP-activated protein kinase activation suppresses leptin expression independently of adipogenesis in primary murine adipocytes

**DOI:** 10.1042/BCJ20240003

**Published:** 2024-02-23

**Authors:** Sophia Bustraan, Jane Bennett, Chad Whilding, Betheney R. Pennycook, David Smith, Alexis R. Barr, Jon Read, David Carling, Alice Pollard

**Affiliations:** 1 Institute of Clinical Sciences, Faculty of Medicine, Imperial College London, London, U.K.; 2 Medical Research Council Laboratory of Medical Sciences, London, U.K.; 3 Emerging Innovations Unit, Discovery Sciences, R&D, AstraZeneca, Cambridge, U.K.; 4 Mechanistic and Structural Biology, Biopharmaceuticals R&D, AstraZeneca, Cambridge, U.K.

**Keywords:** adipogenesis, AMPK, leptins, lipogenesis

## Abstract

Adipogenesis, defined as the development of mature adipocytes from stem cell precursors, is vital for the expansion, turnover and health of adipose tissue. Loss of adipogenic potential in adipose stem cells, or impairment of adipogenesis is now recognised as an underlying cause of adipose tissue dysfunction and is associated with metabolic disease. In this study, we sought to determine the role of AMP-activated protein kinase (AMPK), an evolutionarily conserved master regulator of energy homeostasis, in adipogenesis. Primary murine adipose-derived stem cells were treated with a small molecule AMPK activator (BI-9774) during key phases of adipogenesis, to determine the effect of AMPK activation on adipocyte commitment, maturation and function. To determine the contribution of the repression of lipogenesis by AMPK in these processes, we compared the effect of pharmacological inhibition of acetyl-CoA carboxylase (ACC). We show that AMPK activation inhibits adipogenesis in a time- and concentration-dependent manner. Transient AMPK activation during adipogenic commitment leads to a significant, ACC-independent, repression of adipogenic transcription factor expression. Furthermore, we identify a striking, previously unexplored inhibition of leptin gene expression in response to both short-term and chronic AMPK activation irrespective of adipogenesis. These findings reveal that in addition to its effect on adipogenesis, AMPK activation switches off leptin gene expression in primary mouse adipocytes independently of adipogenesis. Our results identify leptin expression as a novel target of AMPK through mechanisms yet to be identified.

## Introduction

Obesity and associated metabolic diseases are among the leading causes of death worldwide [1]. In England in 2021, ∼64% of adults were estimated to be overweight, including ∼26% classified as obese [2], with obesity estimated tocost the NHS over £6 billion a year [3]. Significant advances in anti-obesity/diabetes treatments, such as the glucagon-like peptide-1 receptor agonist semaglutide (commonly known as Ozempic, Rybelsus, Wegovy) [4,5] and improvements in bariatric surgery outcome [6], have been made in recent years, with promising results. Nevertheless, these treatment options remain expensive, inaccessible to many and responsive, rather than preventative.

Adipose tissue is central to the regulation of whole organism energy homeostasis, and adipose tissue dysfunction is often a driving factor in the progression of overweight to obese, and from obese to other pathologies such as type 2 diabetes, cardiovascular disease and non-alcoholic fatty liver disease [7–10]. Adipose tissue expansion provides a natural buffer to combat overnutrition, allowing excess energy to be stored in the form of triglycerides. As nutrient demand increases, these stores are released through lipolysis to re-enter the circulation and provide fuel for energy-consuming pathways in peripheral tissues. In addition to energy storage, white adipose tissue (WAT) is responsible for the production of hormones, or adipokines, such as leptin and adiponectin, required for organismal energy homeostasis [10–14].

Adipose tissue expands through both adipocyte hypertrophy and by hyperplasia through recruitment of adipose-resident stem cells [15–17]. Found in the perivascular regions [18–20] and in the more recently defined reticular interstitium [21], adipose-derived stem cells (ADSCs) are multi-potent, self-renewing and responsive to peripheral and local stimuli. The maintenance of these properties is imperative for response not only to nutritional excess, but also for the generation of thermogenic adipocytes through adrenergic stimulation [22–24], for the sensitivity to insulin and for the maintenance of pro/anti-inflammatory immune cell responses [15,16,25]. During excessive adipose tissue expansion, ADSCs are subjected to increased mechanical [26,27], hormonal and metabolic perturbations, now accepted as initiating factors contributing to adipose tissue remodelling and, ultimately, dysfunction. Impairment of complete or correct adipogenesis is now thought to contribute to this process [28,29]. Deeper understanding of key signalling pathways disrupted during these processes in adipose stem cells will assist in the development of targeted therapeutics for the treatment and prevention of adipose tissue dysfunction.

AMP-activated protein kinase (AMPK), an evolutionarily conserved heterotrimeric serine/threonine kinase, is widely regarded as a master regulator of energy homeostasis [30–32]. AMPK is central to many metabolic signalling pathways, coordinating catabolism to replenish intracellular ATP, acutely halting anabolic processes and, with chronic activation, contributes to cellular remodelling to promote ATP generation through mitochondrial and lysosomal biogenesis.

Previous studies have associated AMPK activation with the inhibition of adipogenesis, acting in part through the repression of lipogenesis [33–35]. AMPK directly phosphorylates ACC1 at serine 79, preventing the conversion of acetyl-CoA to malonyl-CoA, inhibiting de novo lipid synthesis. In addition, AMPK activation has been associated with a reduction in pro-adipogenic transcription factors such as C/EBPβ and SREBP1-c and their associated targets [36,37]. However, most studies to date achieve AMPK activation using indirect activators, including but not limited to AICAR [38], metformin [39], natural polyphenols and other mitochondrial poisons that increase AMP : ATP ratios. Under these conditions, other pathways are likely to be affected, confounding interpretation of the effects.

Our previous work using an AMPK gain-of-function mouse model, achieving constitutive 2–3-fold activation [40,41], identified a role for in the improvement of adipocyte metabolism and subsequent protection from diet-induced obesity, but there was no obvious inhibition of adipogenesis [41]. To further explore the mechanisms underlying the change in adipocyte metabolism, we decided to evaluate the effect of direct pharmacological AMPK activation on adipogenesis in primary murine ADSCs (mADSCs) *in vitro*.

In this study we employ the direct AMPK activator BI-9774 [42] in a concentration-dependent manner, both throughout differentiation and via a ‘pulse’ treatment during adipogenic initiation. In parallel, we examine the effect of pharmacological inhibition of ACC to determine which, if any, of the effects of AMPK activation on adipogenesis are mediated through ACC inhibition.

## Materials and methods

### Materials

All solvents and chemicals were obtained from Merck (Dorset, U.K.) unless otherwise stated. All cell culture medium was obtained from Gibco (U.K.) unless otherwise stated. 4–12% Bis-Tris gels, first-strand buffer (Invitrogen), SuperScript II Reverse Transcriptase, TRIzol, LipidTOX green neutral lipid stain, TGF beta-1 enzyme-linked immunosorbent assay (ELISA) Kit and Click-iT (5-ethynyl-2′-deoxyuridine) EdU Alexa Fluor 488 HCS Assay were obtained from Invitrogen. Tris-base, acetate, chloroform, EDTA, glycerol, KCl, NaOH, Tris–HCl and Tween-20 were obtained from VWR (West Sussex, U.K.). SensiMix SYBR Hi-ROX was obtained from Bioline (London, U.K.).

#### Primary antibodies (1/1000)

anti-ACC, (Milipore, #05-1098) anti-p-ACCSer79 (CST, #3661), anti-AMPKα (CST, #2793) anti-p-AMPKαThr172 (CST, #2535) anti-β-catenin (CST #8480), anti-CKB (Abclonal, #A12631), anti-CMKT2 (Abcam, #55963), anti-ERK (CST, #4696), anti-p-ERKThr202/Tyr204 (CST, #9101), anti-FAS (CST, #3180), anti-GAPDH (Abcam, #9485), anti-HSL (CST, #4107), anti-HSLSer563 (CST, #4139), anti-Perilipin1 (Abcam, #3526), anti-PPARγ (CST, #2435), anti-SERCA1 (Abcam, #2819), anti-SERCA2 (Invitrogen, #MA3-919), anti-SMAD2/3 (CST, #8685), anti-p-SMAD2Ser465/467 (CST, #3108), anti-p-SMAD3Ser423/425 (CST, #9520), anti-UCP1 (Abcam, #10983), anti-ULK1 (Sigma, #A7481), anti-p-ULK1Ser555 (CST #5869), anti-Vinculin (Sigma, #V9131).

#### Secondary antibodies

Anti-Mouse IRDye® 800CW, (1/20000, Li-Cor Biosciences, #926-32210), anti-Rabbit IRDye® 680RD, (1/20 000, Li-Cor Biosciences, #926-68071), anti-Rabbit HRP-linked, (1/1000, CST, #7074), anti-Rabbit Alexa Fluor 647 (1/1000, Invitrogen, #A-31573), anti-Mouse Alexa Fluor 647, (1/1000, Invitrogen, #A-31571).

### Animal studies

All *in vivo* studies were performed in accordance with the UK Animals (Scientific Procedures) Act (1986) and approved by the Animal Welfare and Ethical Review Board at Imperial College London. All animal experiments were performed at the MRC London Laboratory of Medical Sciences, Hammersmith Hospital, London. All animals used in this study were male C57BL/6J (Charles River, U.K.). Animals were housed in individually ventilated cages at 21°C, with ad libitum access to food and water and 12-h light/ dark cycles. Mice were maintained on an RM3 chow diet (Special Diet Services, Essex U.K.), with a nutrient profile of 11.48% energy from oil, 27.28% from protein and 61.24% from carbohydrate. For all studies mice between 3 and 4 months of age were culled using carbon dioxide euthanasia followed by cervical dislocation.

### Primary adipose tissue stem cell culture

Inguinal WAT depots were dissected and placed in sterile Hank's balanced salt solutions for cell isolation. To isolate mouse inguinal WAT (iWAT) stromal-vascular fraction (SVF) for cell culture, iWAT fat pads were dissected and minced. Minced fat pads were placed in digestion medium (collagenase I (1 mg/ml), collagenase II (1 mg/ml)/dispase II (2 mg/ml)) rotated for 40 min at 37°C with pipetting up and down every 10 min until homogeneous. Digests were passed through a 100 μm filter and centrifuged at 250 × ***g*** for 5 min to pellet the SVF and floating adipocytes were removed with a wide mouth pipette. The SVF pellet was resuspended in 1× red blood cell lysis buffer (BioLegend, London, U.K.) and incubated at room temperature for 4 min. Red blood cell lysis buffer was quenched in phosphate-buffered saline (PBS) and the SVF was pelleted by centrifugation at 250 × ***g*** for 5 min. The SVF was resuspended in SVF medium (10% (v/v) FBS, 1 mM sodium pyruvate, 2 mM l-glutamine, 1% (v/v) penicillin-streptomycin), counted and seeded at appropriate densities. For adipogenic differentiation of primary mouse SVF, cells at 80% confluence were placed into adipogenic induction medium (SVF medium, 1 μg/ml Insulin, 3 nM T3, 0.25 μM dexamethasone, 0.5 mM IBMX, 0.2 mM indomethacin) for 2 days, after which media was exchanged for adipogenic maintenance medium (SVF medium, 1 μg/ml Insulin, 3 nM T3). Media was refreshed every 2–3 days and with mature adipocytes present from day 7 onwards.

### Protein isolation

Protein was isolated from cells by washing with ice cold PBS followed by rapid lysis in ice cold buffer (50 mM HEPES, pH 7.5, 50 mM NaF, 5 mM Na pyrophosphate, 1 mM EDTA, 10% glycerol (v/v), 1 mM dithiothreitol, 4 μg/ml trypsin inhibitor, 0.1 mM phenylmethylsulphonyl fluoride, 175 μg/ml benzamidine, 1% (v/v) Triton X-100. Cells were centrifuged at 13 000 rpm for 10 min at 4°C to remove insoluble material. Protein content of the supernatant was quantified using the Bradford assay against a standard curve. To separate the cytosolic and nuclear fractions, cells were washed in ice cold PBS and rapidly lysed and scraped into cytosolic protein extraction buffer (10 mM MES pH 6.5, 5 mM MgCl_2_, 1 mM CaCl_2_, 15 mM NaCl, 60 mM KCl, 0.25 M sucrose, 0.5% (v/v) Triton X-100, 0.5 mM sodium metabisulfite, 1 mM DTT, 4 μg/ml trypsin inhibitor, 0.1 mM phenylmethylsulphonyl fluoride, 175 μg/ml benzamidine) and left on ice for 15 min. Cells were centrifuged at 3600 rpm for 10 min at 4°C and the supernatant containing the cytosolic proteins was collected. To remove any excess cytosolic proteins, the remaining pellet was resuspended in cytosolic protein extraction buffer again and left on ice for 15 min before centrifuging at 3600 rpm for 10 min at 4°C, this time discarding the supernatant. The pellet was resuspended in nuclear extraction buffer (20 mM Tris–HCl pH 7.9, 10 mM EDTA, 300 mM NaCl) and sonicated with a probe sonicator for 10 s to release the nuclear proteins. Then, both the cytosolic and nuclear fractions were centrifuged at 13 000 rpm for 10 min at 4°C and the supernatants collected.

### Western blotting

1 μg/μl protein lysate in sample buffer was heated to 100°C for 5 min, except when mitochondrial complex proteins were being analysed, in which case samples were not heated. Following SDS–PAGE, samples were transferred to polyvinylidene fluoride membrane. The membrane was blocked by incubation in buffer containing 4% (w/v) BSA in tris-buffered saline containing 0.1% (v/v) Tween20 (TBS-T) for 1 h. Blots were incubated with primary antibodies in 4% (w/v) BSA overnight at 4°C then washed three times in TBS-T. Blots were incubated with secondary antibodies in 4% (w/v) milk in TBS-T for 1 h at room temperature and then washed three times in TBS-T. Blots were imaged with an Odyssey Imaging System (LI-COR Biosciences) and quantified using Image Studio Lite Version 5.2.

### Immunofluorescence

Cells were washed with PBS and then fixed in 4% paraformaldehyde plus 2% sucrose in PBS for 10 min at room temperature. Cells were washed three times with PBS. Cells were blocked and permeabilised with immunofluorescence blocking buffer (1% (v/v) BSA, 0.1% Triton X-100) for 1 h at room temperature. Cells were incubated with primary antibody in immunofluorescence blocking buffer overnight at 4°C. Cells were washed three times with PBS and incubated with secondary antibodies in immunofluorescence blocking buffer for 1 h at room temperature followed by three PBS washes. For lipid droplet staining, cells were incubated with 1/500 LipidTOX dye dissolved in PBS for 30 min at room temperature in the dark. Cells were washed in PBS and then incubated with 1 μg/ml DAPI for 5 min in the dark at room temperature and then put into fresh PBS. Whole well scans were acquired on the EVOS M700 and quantification of neutral lipid stain intensity and nuclei counts were performed with ImageJ software. Adipogenic index was calculated as a ratio of neutral lipid stain intensity to nuclei counts. Images of EdU and PPARγ stained cells were acquired on the Yokogawa microscope and staining was quantified with Columbus software. Images for lipid droplet size quantification were acquired on the Operetta microscope and lipid droplet size was quantified with Harmony software (PerkinElmer). To determine lipid droplet size, first nuclei were segmented based on DAPI intensity. Next, cells containing lipid droplet were identified based on LipidTOX intensity in a six-pixel ring surrounding the nuclei. Whole cell segmentation of LipidTOX positive nuclei was then performed based on the intensity of the LipidTOX marker. Individual lipid droplets within LipidTOX positive segmented cells were identified using the SpotFinder function and spot morphology features were extracted.

### Edu labelling

Before fixing with paraformaldehyde as described in 2.5, cells were labelled with 10 μM EdU for 30 min. Fixed cells were blocked and permeabilised with immunofluorescence blocking buffer for 1 h at room temperature. To detect EdU the Click-iT EdU Assay kit was used according to the manufacturer's instructions. The Click-iT EdU reaction cocktail containing EdU reaction buffer, CuSO_4_, Alexa Fluor 488 Azide and buffer additive was made immediately before use. The cells were incubated with the Click-iT EdU reaction cocktail for 30 min at room temperature in the dark and then washed three times in the immunofluorescence blocking buffer.

### RNA isolation

RNA was isolated from cells using TRIzol plus 0.4%(v/v) chloroform. The mix was shaken for 15 s then incubated for 2 min at room temperature before being centrifuged at 12 000 × ***g*** for 10 min at 4°C. The clear aqueous phase was taken and mixed with 0.53 vol ethanol and RNA was purified with an RNeasy kit (Qiagen) according to the manufacturer's instructions. RNA was eluted into RNase free water by centrifuging at 12,000 × ***g*** for 1 min. RNA concentrations were measured on a NanoDrop photospectrometer (Thermo Scientific).

### cDNA synthesis and quantitative PCR

1 μg RNA was incubated at 65°C for 10 min with 5 ng/μl random hexamers, 1 mM dNTP mix and RNase free water in a volume of 10 μl. Then, 1× first-strand buffer, 20 mM dithiothreitol, 200U SuperScript™ II Reverse Transcriptase, 10 mM MgCl_2_ and RNase free water was added to the RNA mix to make a final volume of 20 μl and incubated at 25°C for 10 min, 50°C for 50 min, 85°C for 5 min. For quantitative PCR (qPCR), 2 μl cDNA was added to 1× SensiMix SYBR Hi-ROX, 400 nM forward/reverse primer mix (36B4 (forward: AGATTCGGGATATGCTGTTGGC, reverse: TCGGGTCCTAGACCAGTGTTC), Adipoq (forward: AGGCCGTTCTCTTCACCTAC, reverse: CTGGTGTATGGGCTATGGGT), Cebpα (forward: GCGGGAACGCAACAACATC, reverse: GTCACTGGTCAACTCCAGCAC), Fas (forward:TGCTCCCAGCTGCAGGC, reverse: GCCCGGTAGCTCTGGGTGTA), Lep (forward: GAGACCCCTGTGTCGGTTC, reverse: CTGCGTGTGTGAAATGTCATTG), Lpl (forward: ATCGGGCCCAGCAACATTAT, reverse: ACTCCTCCTCCATCCAGTTG), Pparγ (forward: TGAAAGAAGCGGTGAACCAC, reverse: AGCTGATTCCGAAGTTGGTG) and RNase free water to make up a total volume of 8 μl. Reactions were run on a thermal cycler (Bio-Rad CFX) to measure *c*(*t*) values. qPCR primer linearity was evaluated by pooling all cDNA and serial diluting to make a standard curve. To interpret gene expression, the *c*(*t*) values were input into the linear equation *y* = *mx* + *c* generated from the standard curves and *x* was found. This value was then transformed with 10*^x^* to account for the logarithmic scale of the standard curve and then normalised to a housekeeping gene.

### Leptin ELISA

Media was collected from cells and leptin content evaluated using a Leptin ELISA (#KMC2281, Thermo Scientific) according to manufacturer's instructions. Briefly, leptin standards and samples were added to pre-coated wells and incubated for 2 h at 37°C. The plate was washed and further incubated with streptavidin-HRP for 30 min at room temperature. The plate was washed and incubated with chromogen for 30 min. The reaction was stopped and the absorbance at 450 nm quantified. Leptin concentrations were determined using a standard curve plotted with a four-parameter algorithm.

### Statistical analyses

Unless stated otherwise, all data are presented as the mean ± SEM. Differences between two data sets were analysed using a Student's two-tailed *t*-test. *F*-tests were used to compare the variances and where there was a significant difference between the variances (*P* < 0.05), Welch's correction was applied. One-way analysis of variance (ANOVA) was used to assess differences between multiple groups with Dunnett's correction for multiple comparisons when comparing between one control group, or *post hoc* Tukey's test when comparing the differences between all groups. When comparing the differences between two variables with multiple groups per variable, two-way ANOVA was used with Šidák's correction for multiple comparisons. For all, a statistically significant difference was defined as *P* < 0.05. All statistical analysis were performed using GraphPad Prism software version 9.1.

## Results

### Effect of pharmacological activation of AMPK by BI-9774 in undifferentiated mADSCs

**Figure 1.**
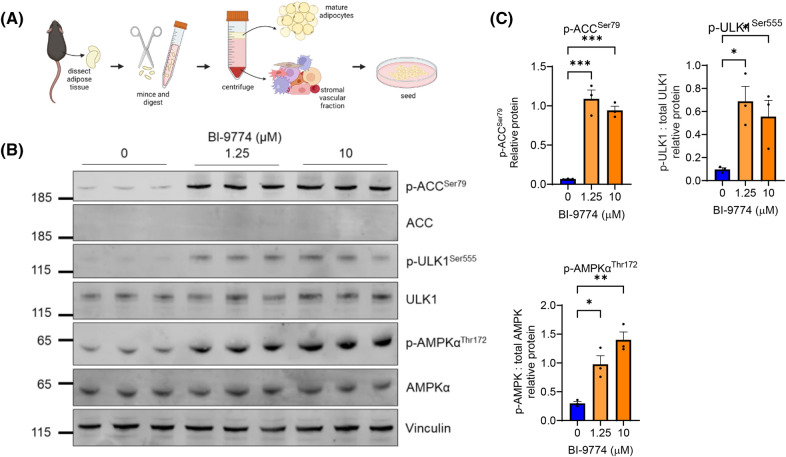
AMPK activation by BI-9774 in ADSCs and mature adipocytes. (**A**) Schematic for the isolation, digestion and culture of primary murine ADSCs (mADSCs). (**B**) Undifferentiated stromal vascular fraction (SVF) was treated with 1.25 or 10 µM BI-9774 for 2 h and AMPK, ACC1 and ULK1 phosphorylation determined by Western blot analysis. (**C**) Quantification of p-ACC^Ser79^ relative to vinculin, p-ULK1^Ser555^ relative to total ULK1 and p-AMPK^Thr172^ relative to total AMPKα. Data are shown as mean ± SEM (*n* = 3) Statistical significance is determined by one-way ANOVA with Dunnett's correction for multiple comparisons, *P < 0.05, **P < 0.01, ****P < 0.0001.

To explore the role of AMPK activation in primary mADSCs, subcutaneous adipose tissue was excised, digested and the SVF isolated via centrifugation (Figure 1A). To confirm target engagement, undifferentiated ADSCs were treated with BI-9774 for 2 h and phosphorylation of Thr172 on AMPK and of downstream targets of AMPK was assessed. Increased phosphorylation of ACC^Ser79^ and ULK1^Ser555^ was achieved with 1.25 µM BI-9774, concurrent with an increase in phosphorylation of AMPKα^Thr172^ (Figure 1B,C). Total ACC was below the level of detection in undifferentiated cells due to antibody sensitivity. Therefore, phosphorylation of ACC was normalised to vinculin level in Figure 1B. No significant difference in target phosphorylation was observed between 1.25 and 10 µM BI-9774 treatment, suggesting maximal phosphorylation of these targets at 1.25 µM compound.

### Effect of pharmacological activation of AMPK by BI-9774 in mature adipocytes

**Figure 2.**
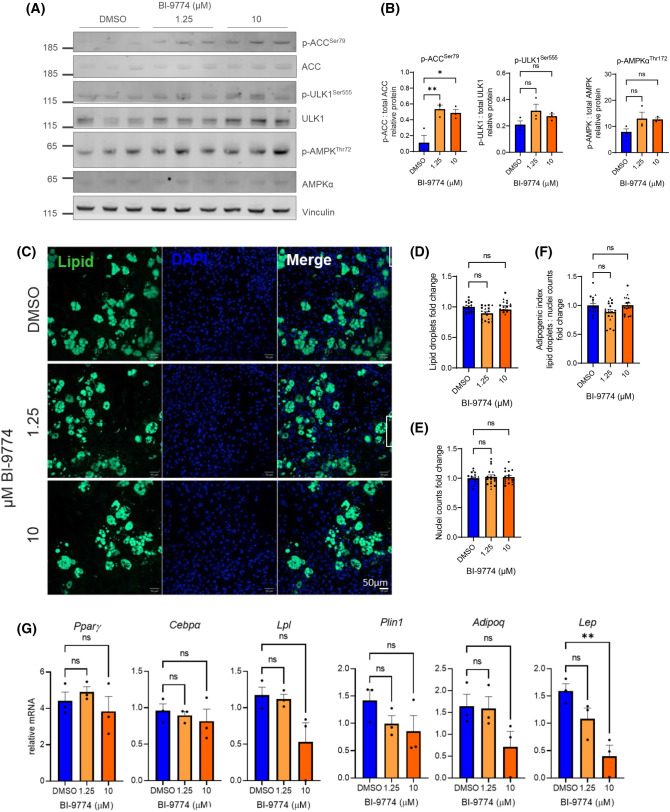
Activation of AMPK in mature adipocytes suppresses leptin independently of lipid accumulation and transcription factor expression. (**A**) Mature SVF-derived adipocytes were treated with 1.25 or 10 µM BI-9774 for 3 days and AMPK, ACC1 and ULK1 phosphorylation determined by Western blot analysis. (**B**) Quantification of p-ACC relative to vinculin, p-ULK1 relative to total ULK1 and p-AMPK relative to AMPKα. Statistical significance is determined by one-way ANOVA with Dunnett's correction for multiple comparisons, *P < 0.05, **P < 0.01, ***P < 0.001 (*n* = 3). (**C**) Representative images of whole well scans of mature adipocytes treated with either 1.25 or 10 µM BI-9774 for 3 days, with lipid droplet and DAPI staining. Quantification of (**D**) lipid droplet staining (LipidTOX 488 nm), (**E**) nuclei counts (DAPI) and (**F**) adipogenic index (ratio of lipid droplet number: nuclei). Data are shown as mean ± SEM (*n* = 3), with biological replicates represented by different shaped symbols. Statistical significance is determined by one-way ANOVA with Dunnett's correction for multiple comparisons, *P < 0.05, ****P < 0.0001. Scale bar: 1500 μm. (**G**) Adipocyte marker mRNA expression in mature adipocytes with or without BI-9774 treatment for 3 days, relative to housekeeper gene 36B4. Data are shown as mean ± SEM (*n* = 3), with statistical significance determined by one-way ANOVA with Dunnett's correction for multiple comparisons, **P < 0.01.

To evaluate target engagement in mature adipocytes, cells were differentiated for 8 days. At maturity, adipocytes were treated with 1.25 or 10 µM BI-9774 for a further 3 days. A significant increase in phosphorylation of ACC^Ser79^ relative to total ACC, both relative to vinculin, was observed at both concentrations, with maximal phosphorylation achieved at 1.25 µM (Figure 2A,B). In contrast, no significant increase in phosphorylation of ULK1^Ser555^ or AMPKα^Thr172^ was observed, though a significant decrease in both total AMPKα and ULK1 was noted in mature adipocytes in comparison with undifferentiated ADSCs (Figure 2A,B). No difference in lipid droplet accumulation (LipidTOX neutral lipid stain) (Figure 2C,D) nuclei count (Figure 2E) or adipogenic index (Figure 2F) was observed. A significant reduction in leptin gene expression (*Lep*) at 10 µM BI-9774 (Figure 2G) was observed, with a trending decrease at 1.25 µM. No significant effect on expression of any other adipocyte marker genes (*Pparg*, *Lpl*, *Plin1*, *Adipoq*) was identified under these conditions. No effect of AMPK was observed on either canonical (UCP1) or non-canonical (creatine kinase B; CKB, SERCA2) thermogenic pathways in unstimulated (Supplementary Figure S1A) or 10 µM isoprenaline-stimulated (Supplementary Figure S1B) mature adipocytes with either 1.25 or 10 µM BI-9774. Neither creatine kinase (CKMT2) nor SERCA1 were detected at protein level in mature adipocytes in either condition.

### Chronic AMPK activation reduces adipogenesis and lipid droplet size in a concentration-dependent manner

**Figure 3.**
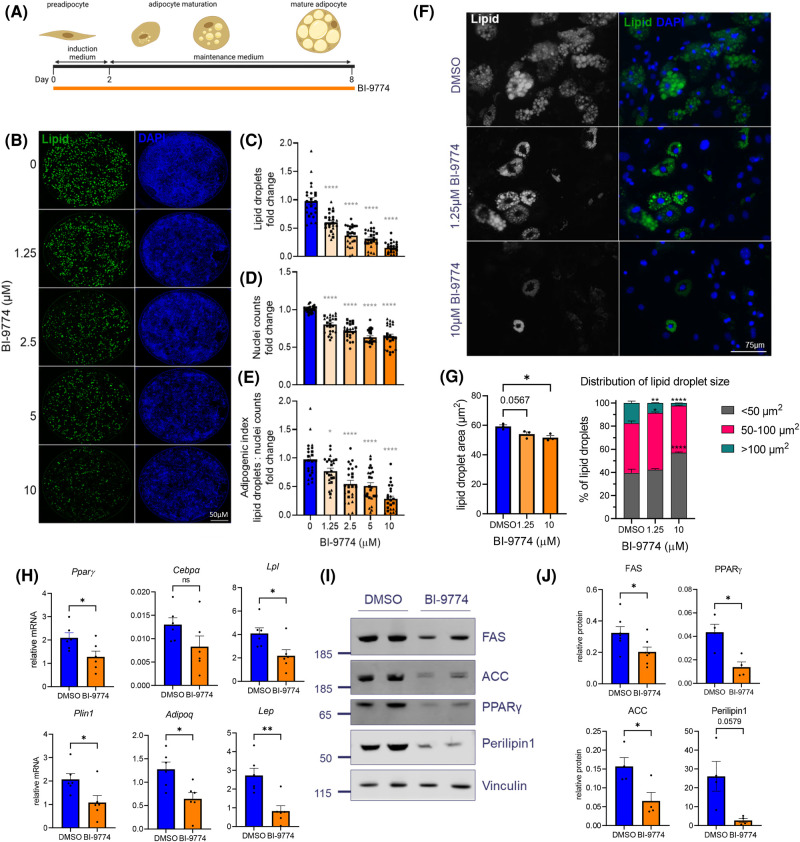
Chronic AMPK activation reduces adipogenesis and lipid droplet size in a concentration-dependent manner. (**A**) Schematic depicting the culture and differentiation of murine ADSCs into mature adipocytes. Day 0 (2 days post-isolation) cells were switched to adipogenic induction medium, +/− BI-9774 at indicated concentrations. After 2 days, cells were transferred into maintenance media (+/− BI-9774) and maintained for a further 6 days. (**B**) Representative images of whole well scans with lipid droplet and DAPI staining. Quantification of (**C**) lipid droplet staining (LipidTOX 488 nm), (**D**) nuclei counts (DAPI) and (**E**) adipogenic index. Data are shown as mean ± SEM (*n* = 3), with biological replicates represented by different shaped symbols. Statistical significance is determined by one-way ANOVA with Dunnett's correction for multiple comparisons, *P < 0.05, ****P < 0.0001. Scale bar: 1500 μm. (**F**) Representative image of lipid droplets and (**G**) Mean average lipid droplet size and the distribution of lipid droplet size at 1.25 and 10 µM BI-9774. Data are shown as mean ± SEM (*n* = 3), one-way or two-way ANOVA with Dunnett's correction for multiple comparisons, *P < 0.05, **P < 0.01, ****P < 0.0001. (**H**) Adipocyte marker mRNA expression at the end of SVF adipogenic differentiation with or without 1.25 µM BI-9774. mRNA levels shown relative to housekeeper gene 36B4. Data are shown as mean ± SEM (*n* = 6), and statistically significant differences determined using Student's *t*-test, *P < 0.05, **P < 0.01. (**I**) Representative Western blot and (**J**) quantification of adipocyte associated proteins with quantification for FAS, ACC, PPARγ and perilipin 1 relative to vinculin. Data are shown as mean ± SEM (*n* = 4), Student's two tailed *t*-test with Welch's correction applied for Perilipin1, *P < 0.05.

To evaluate the concentration-dependent effects of AMPK activation on lipid accumulation, mADSCs were differentiated for 8 days in the absence or presence of 1.25, 2.5, 5 and 10 µM BI-9774 (Figure 3A). Using imaging analysis (Figure 3B), a significant reduction in lipid droplet staining (LipidTOX neutral lipid stain) was observed in response to low-concentration (1.25 µM) BI-9774 treatment. A further, concentration-dependent reduction in lipid accumulation was observed between 1.25 and 10 µM BI-9774 (Figure 3C). In addition to a reduction in lipid accumulation, a concentration-dependent reduction in nuclei counts was observed between 1.25 and 5 µM BI-9774. No significant difference in nuclei count was observed between 5 and 10 µM BI-9774 (Figure 3D). To evaluate the effect of AMPK activation on adipogenesis, the ratio of lipid droplets to nuclei, or adipogenic index, was determined (Figure 3E). Together these findings suggest AMPK activation reduces adipogenesis in a concentration-dependent manner, with mild reduction observed at 1.25 µM and robust decrease at 10 µM BI-9774.

To establish the effects of AMPK activation on lipid droplet size, images from cells treated with either low (1.25 µM) or high (10 µM) BI-9774 were analysed using high throughput imaging analysis (Harmony). Both low and high concentration BI-9774 led to a reduction in lipid droplet size (Figure 3F), although this decrease only reached statistical significance at 10 µM BI-9774. The percentage of lipid droplets >100 µm^2^ was significantly reduced by low and high concentrations of BI-9774, with a shift towards those <50 µm^2^ at 10 µM BI-9774 (Figure 3G).

In addition to a reduction in lipid accumulation and droplet size, chronic AMPK activation by BI-9774 at 1.25 µM led to a reduction in adipocyte-associated gene expression (*Pparg*, *Lpl*, *Plin1*, *Adipoq*, *Lep*) (Figure 3H) and protein levels (ACC1, fatty acid synthase (FAS) and perilipin 1 (PLIN1)) (Figure 3I,J). To determine the contribution of adipocyte number to total adipogenic index, PPARγ^+ve^ nuclei were identified by immunofluorescence and EdU incorporation was performed in tandem to identify proliferating cells at the end of differentiation (Supplementary Figure S2A). Low-concentration chronic AMPK activation significantly reduced PPARγ^+ve^ nuclei counts and increased EdU^+ve^ cell staining, with a reduction in total nuclei count (Supplementary Figure S2B). No significant differences were observed in adipogenic transcription factor Srebp (*Srebf1*, *Srebf2*) mRNA expression (Supplementary Figure S2C). Previous studies have suggested AMPK activation mediates response to adrenergic receptor stimulation in adipocytes. To address this, adipocytes were differentiated for 8 days in the presence/absence of 1.25 µM BI-9774, then stimulated for 24 h with the non-selective β-adrenoceptor agonist isoprenaline (10 µM). No alteration in basal or stimulated phosphorylation of hormone sensitive lipase (HSL) at serine 563 was observed between DMSO and BI-9774 treated cells, as shown by Western blotting (Supplementary Figure S2D,E).

AMPK activation by 5-aminoimidazole-4-carboxamide riboside (AICAR) and A-769662 has been shown to repress adipogenesis, in part, through transforming growth factor (TGF)β/wingless (Wnt) signalling pathways, outlined in Supplementary Figure S3A [43,44]. We therefore evaluated the effects of AMPK activation by BI-9774 on SMAD2/3 phosphorylation and translocation of β-catenin in SVF after 2 days of adipogenic induction and at the end of adipogenesis, in the presence/absence of BI-9774. A significant reduction in TGFβ protein was observed in cell lysate (Supplementary Figure S3B), concurrent with a modest but significant increase in TGFβ2 mRNA expression (Supplementary Figure S3C) however no significant differences in TGFβ-ERK-SMAD signalling were observed (Supplementary Figure S3D,E) at either time-point. To assess the impact of AMPK activation on Wnt signalling, translocation of SMAD proteins and β-catenin to the nucleus was assessed using cytosolic and nuclear cell extracts at 2 days post-induction and in mature adipocytes in the presence/absence of AMPK activation. Translocation of all proteins was observed after 8 days of adipogenesis. No differences in β-catenin translocation were observed (Supplementary Figure S3F,G) at either time-point with 1.25 µM BI-9774.

### Activation of AMPK during adipocyte maturation suppresses leptin expression independent of adipogenesis

**Figure 4.**
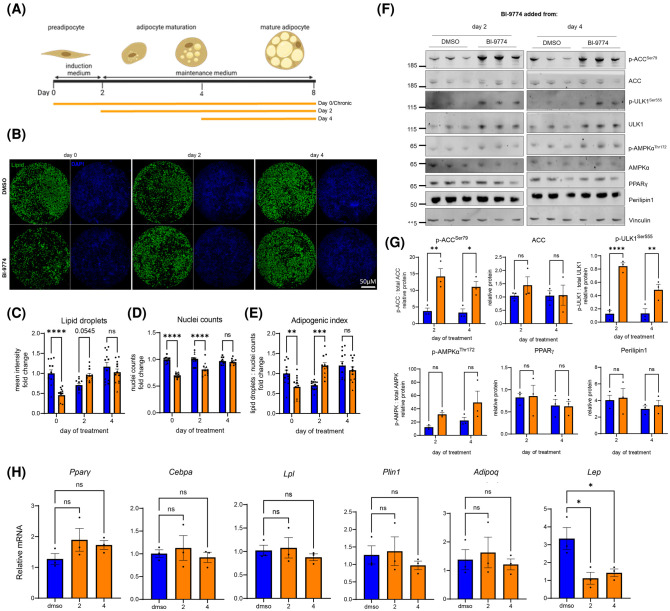
Suppression of Leptin by AMPK activation is independent of adipogenesis. (**A**) Schematic to illustrate time-course of the addition of BI-9774 (1.25 µM) treatment during adipogenic maturation. Day 2 and 4 indicate 2- and 4-days post induction, with BI-9774 added to maintenance medium and retained until termination. (**B**) Representative images of whole well scans with lipid droplet and DAPI staining. Quantification of (**C**) lipid droplet staining (LipidTOX 488 nm) (**D**) nuclei counts (DAPI) and (**E**) adipogenic index. Data are shown as mean ± SEM (*n* = 3), with biological replicates represented by different shaped symbols. Statistical significance is determined by one-way ANOVA with Dunnett's correction for multiple comparisons, *P < 0.05, ****P < 0.0001. Scale bar: 1500 μm. (**F**) Representative western blot and (**G**) Quantification of p-ACC^Ser79^ relative to total ACC, p-ULK^Ser555^ relative to total ULK1, p-AMPKα^Thr172^ relative to AMPKα. PPARγ and Perilipin 1 levels are relative to vinculin. Data are shown as mean ± SEM with statistical significance determined by two-way ANOVA with Šidák's correction for multiple comparisons, *P < 0.05, **P < 0.01, ****P < 0.0001, (*n* = 3). (**H**) Adipocyte marker mRNA expression at the end of SVF adipogenic differentiation with or without 1.25 µM BI-9774. mRNA expression relative to housekeeper gene 36B4. Data are shown as mean ± SEM (*n* = 3), and statistically significant differences determined using one-way ANOVA with Dunnett's correction for multiple comparisons, *P < 0.05.

To determine the effects of AMPK on adipocyte maturation, and to assess whether the suppression of leptin by AMPK was solely due to a reduction in adipogenesis, ADSCs were induced in the absence of BI-9774. After 2 days induction, cells were transferred into maintenance medium with or without 1.25 µM BI-9774. After 2 days, a second timepoint was initiated, with BI-9774 added on day 4 (2 days after maintenance medium) (Figure 4A). At the end of differentiation, cells were imaged (Figure 4B) to assess adipogenesis by lipid accumulation (Figure 4C) nuclei counts (Figure 4D) and adipogenic index (Figure 4E). AMPK activation from day 2, unlike day 0, significantly increased the adipogenic index. Activation from day 4, akin to effects in mature cells, did not affect adipogenesis, despite significant AMPK activation as assessed by phosphorylation of ACC and ULK1. Despite an increase in adipogenic index at day 2, no significant effects of AMPK activation were observed on perilipin 1 or PPARγ protein levels (Figure 4F,G). No effects were observed on adipogenic gene expression when BI-9774 was added from either day 2 or 4 (Figure 4H), however a significant reduction in leptin mRNA was observed, indicating that AMPK suppresses leptin independent of adipogenesis.

### Short-term ‘pulse’ AMPK activation in mADSCs is sufficient to reduce adipogenesis and suppress leptin expression

**Figure 5.**
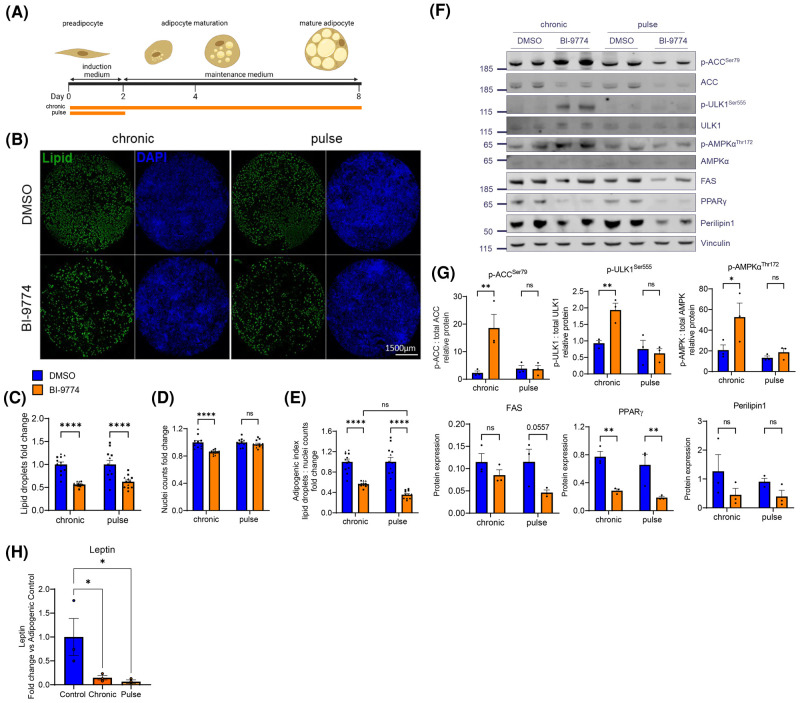
Transient AMPK activation is sufficient to reduce adipogenesis and repress leptin expression in mADSCs. (**A**) Schematic depicting the culture and differentiation of murine ADSCs into mature adipocytes with either chronic or ‘pulse’ (induction period only) treatment with 1.25 µM BI-9774. (**B**) Representative images of whole well scans with lipid droplet and DAPI staining. Quantification of (**C**) lipid droplet staining (**D**) nuclei counts and (**E**) adipogenic index. Data are shown as mean ± SEM (*n* = 3), with biological replicates represented by different shaped symbols. Statistical significance is determined by two-way ANOVA with Šidák's correction for multiple comparisons, ****P < 0.0001. Scale bar: 1500 μm. (**F**) Representative Western blot and (**G**) quantification of protein levels in response to chronic or pulse-treatment with BI-9774. Protein phosphorylation is shown relative to the corresponding total protein; adipocyte associated proteins (FAS, PPARγ and Perilipin1) are shown relative to vinculin level. Data are shown as mean ± SEM (*n* = 3) with statistical significance determined by two-way ANOVA with Šidák's correction for multiple comparisons, *P < 0.05, **P < 0.01. (**H**) Leptin protein levels in cell culture supernatant as determined by ELISA. Data are shown as mean ± SEM (*n* = 3 biological replicates), one-way ANOVA, *P < 0.05.

Chronic AMPK activation is known to repress adipogenesis, though due to the large number of AMPK targets affected during this process, the specific mechanism by which this occurs is difficult to delineate. To establish the effects of AMPK on the initiation of adipogenesis, mADSCs were treated with 1.25 µM BI-9774 for 2 days, in adipogenic induction medium. This transient ‘pulse’ treatment was then removed following transition to adipogenic maintenance medium. The media was changed every 2 days, removing any remaining compound (Figure 5A). Surprisingly, we found that lipid accumulation was equally reduced by pulse and by chronic AMPK activation (Figure 5B,C). Transient AMPK activation did not affect nuclei counts (Figure 5D) and reduced the adipogenic index in a manner comparable to chronic AMPK activation (Figure 5E).

Following removal of BI-9774, no sustained AMPK activation by pulse treatment was observed after 8 days of differentiation, as determined by phosphorylation of AMPKα^Thr172^, ACC^Ser79^ and ULK1^Ser555^ (Figure 5F,G). Consistent with the adipogenic index, a significant decrease in protein expression of the adipogenic transcription factor PPARγ was observed, though no significant reduction in adipogenic protein levels was observed (Figure 5G). To further explore the effects of AMPK activation on leptin expression, media collected from cells cultured with either pulse or chronic AMPK activation during adipogenesis was evaluated for total leptin protein by ELISA. Consistent with transcriptional data, media-leptin content, though variable in untreated samples, was significantly and consistently reduced by both chronic and pulse AMPK activation (Figure 5H).

### The regulation of adipogenesis by transient AMPK activation is independent of ACC

**Figure 6.**
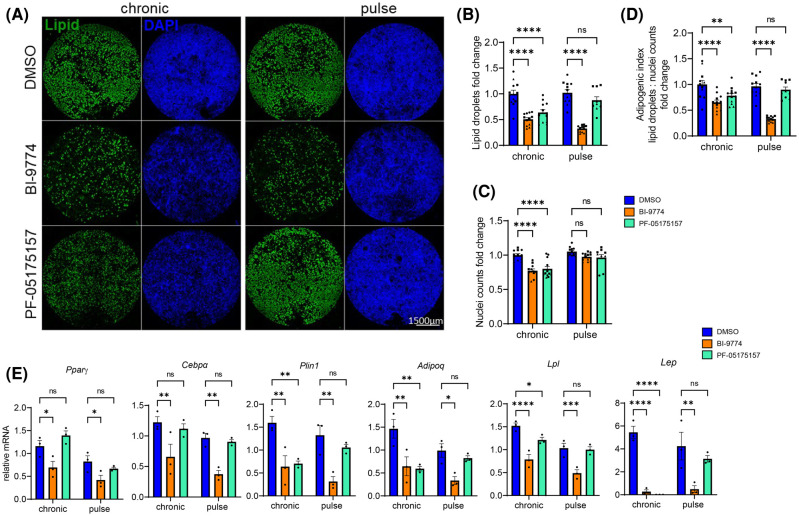
The effects of transient AMPK activation on adipogenesis are independent of ACC inhibition. To compare the effects of AMPK activation on adipogenesis to ACC inhibition, cells were treated either transiently or chronically treated with 1.25 μM BI-9774 or 5 μM ACC-inhibitor PF-051575157. (**A**) Representative images of whole well scans with lipid droplet and DAPI staining. Quantification of (**B**) lipid droplet staining (**C**) nuclei counts and (**D**) adipogenic index. Data are shown as mean ± SEM (*n* = 3), with different biological replicates represented by different shaped symbols. Statistical significance is determined by two-way ANOVA with Šidák's correction for multiple comparisons, **P < 0.01, ****P < 0.0001. Scale bar: 1500 μm. (**E**) Adipocyte marker mRNA expression levels relative to housekeeper gene 36B4. Data are shown as mean ± SEM (*n* = 3), two-way ANOVA with Šidák's correction for multiple comparisons, *P < 0.05, **P < 0.01, ***P < 0.001, ****P < 0.0001.

To determine whether the observed reduction in adipogenesis upon AMPK activation was due to the inhibition of ACC, cells were treated with either 1.25 µM BI-9774 or the ACC inhibitor PF-05175157 administered chronically, or as a pulse treatment as described previously. Chronic ACC inhibition (Figure 6A) was sufficient to reduce the adipogenic index, 5 µM PF-05175157 resulting in equivalent lipid droplet staining (Figure 6B) nuclei count (Figure 6C) and reduction in adipogenic index (Figure 6D) as seen with1.25 µM BI-9774. In marked contrast with transient treatment with BI-9774, there was no significant reduction in adipogenesis following transient treatment with PF-05175157, suggesting that the effects of AMPK during adipogenic initiation cannot be solely attributed to the inhibition of ACC.

Chronic ACC inhibition significantly reduced mature adipocyte-associated gene expression (*Plin1*, *Lpl*, *Adipoq*, *Lep*), but had no significant effect on expression of adipogenic transcription factors (*Pparg*, *Cebpa*)*.* In contrast, both transient and chronic activation of AMPK by BI-9774 resulted in the reduction in both adipogenic transcription factors and terminal adipogenesis markers, most notably an almost complete repression of leptin gene expression (Figure 6E).

Together these data identify a previously unexplored signalling pathway connecting AMPK activation to leptin signalling, independent of ACC inhibition, that cannot be wholly attributed to an inhibition of adipogenesis.

## Discussion

Preservation of adipocyte and ADSC metabolism is integral to the prevention and treatment of metabolic disease. Inhibition of healthy adipose tissue expansion is now recognised as a driving factor for peripheral lipid accumulation, dyslipidemia, hormonal imbalance and chronic, systemic inflammation. Preservation of healthy adipocyte differentiation should therefore take centre-stage in the fight against obesity and the metabolic syndrome. In contrast, most literature focuses on the inhibition of adipogenesis as a possible therapeutic strategy, rather than the preservation of, or improvement, in metabolic function of adipocytes. Many of these studies highlight AMPK as a possible tool for the inhibition of adipogenesis, highlighting its key role in lipid metabolism, and in the control of lipogenic gene expression.

In this study, we set out to interrogate the links between AMPK signalling and adipogenesis in a primary mouse adipose-derived cell culture model. We utilised the highly selective small molecule AMPK activator BI-9774. This minimises the potential off-target effects that can occur when using less-specific AMPK activators that have been used in previous studies exploring the role of AMPK in adipogenesis *in vitro*. Our data show that specific AMPK activation reduces adipogenesis in a concentration-dependent manner, with impact on lipid accumulation, droplet size and on cell number. However, the reduction in total adipogenesis by low-concentration chronic AMPK activation, as determined by PPARγ^+ve^ nuclei, and by adipogenic index, is modest (44%), suggesting AMPK activity does not completely impair adipogenesis under these conditions. The decrease in adipogenesis was not due simply to decreased proliferation as EdU staining was modestly, but significantly, increased.

Lipid droplet formation, and lipid accumulation through lipogenesis are key hallmarks of adipogenesis, both of which are known cascades modulated by AMPK [34,35,45–47]. In this study we therefore sought to delineate the effects of ACC inhibition and AMPK activation during adipogenesis using a direct ACC inhibitor, PF-05175157. Chronic ACC inhibition significantly reduced adipogenesis and lipid droplet size, with a corresponding reduction in adipocyte marker expression. However, unlike chronic AMPK activation, chronic ACC inhibition did not decrease levels of *Pparγ* or *Cebpα*, suggesting that adipocyte differentiation itself is not impaired, rather the maturity of the adipocytes formed is impaired. This result is perhaps unsurprising, as lipogenesis is required not only for the accumulation of lipid droplets, but for the maturation of adipocytes to facilitate the production of adipokines, such as leptin, repressed in this system by both chronic AMPK activation and ACC inhibition.

Adipogenesis requires convergence of many signalling pathways, all resulting in the initiation of transcription factor binding, followed by chromatin remodelling and, ultimately, adipogenic gene expression [8,24]. AMPK is known to interact with and/or modulate the expression of many of these factors, including ChrEBP, SREBP1-c/2, PPARγ and PGC1α, with documented AMPK-dependent repression of translocation, binding and/or expression in each case [42,48–54].

In previous studies, AMPK activation has been shown to inhibit adipogenesis through the TGFβ/Wnt signalling pathway using AICAR [44]. Despite a striking decrease in TGFβ protein expression, we did not observe any effects of specific AMPK activation by BI-9774 on TGFβ/Wnt signalling. These results may vary due to the primary nature of our cell model, or may be due to the off-target effects of AICAR. Indeed, the effect of both AICAR and A-769662, a small molecule AMPK activator, have been shown to modulate glucose uptake in adipocytes in an AMPK-independent manner [55], suggesting the effects of AICAR on TGFβ/Wnt may also be independent of AMPK. Further studies using AMPK knockout models are required to evaluate these differences, and to investigate the role of AMPK in TGFβ/Wnt signalling in adipocytes under inflammatory conditions, where TGFβ signalling is elevated.

Other previous studies in 3T3-L1 preadipocytes have evaluated the effects of AICAR, on adipogenesis, and suggest AMPK activation is only inhibitory between day 0 and day 3 [38]. Additional work in 3T3-L1 using resveratrol analogues as indirect AMPK activators have also noted similar effects in cells, in addition to a decrease in bodyweight in mice fed a high fat diet [56]. To focus on the specific role of AMPK in the early stages of adipogenesis, we employed a low-concentration transient pulse treatment with BI-9774 for the 2 days of adipogenic initiation, followed by a maturation period in the absence of the activator. Despite a lack of ACC/ULK1phosphorylation, or residual AMPK phosphorylation at Thr172, we observed a significant, lasting repression of adipogenic transcription factor (*Cebpα*, *Pparγ*) and adipogenic gene expression that could not be replicated by transient ACC inhibition, as well as a robust inhibition of leptin transcription and protein level. In addition, we show that AMPK may only inhibit adipogenesis if present during commitment to the adipogenic lineage, and if activated beyond this point, its effects on adipogenesis are minimal, save for the striking effects on leptin expression. As far as we know, this is the first study to show an effect of AMPK on leptin expression.

Leptin, produced primarily by adipocytes, regulates food intake, energy expenditure and adiposity [57]. Loss of functional leptin in mice leads to obesity and T2D, driven by hyperphagia and a reduction in energy expenditure [57–60]. Besides genetic ablation/loss of function, upstream regulation of leptin is significantly less studied than its many downstream targets. In 1996, C/EBPα was identified as a transcriptional regulator of the *ob* (leptin) gene [61]. Later, Mason et al. [60], described positive regulation of leptin promotor function C/EBP, specificity protein 1 and lipid transfer protein 1, and a lack of leptin promotor activation in response to PPARγ or SREBP-1 in rat adipocytes. Additional work by the Spiegelman laboratory identified SREBP-1 as a key insulin-responsive trans-activator of leptin gene expression in rat adipocytes [62]. Here we identify CEBPα as an AMPK-responsive, ACC-independent gene during adipocyte commitment, the reduction in which may provide a partial explanation for the reduction in leptin expression in our model. AMPK has also been implicated in the phosphorylation and inhibition of SREBP1-c/2 (Ser372) in cells and in liver [46,47,63], though this remains to be explored under the conditions presented here. No alteration in SREBP (*Srebf1/2*) gene expression was identified in response to AMPK activation, however this does not rule out modulation due to direct transcriptional activity and/or DNA binding.

Though gaining traction in other fields [53,64], the role of AMPK in the epigenetic regulation of adipogenesis is not well understood, and has been historically challenging to separate from the inhibition of lipogenesis/lipid accumulation. In this study we have presented a robust protocol and platform for the study of AMPK and other modifiers of adipogenesis in a primary mouse cell culture model, with use of low-concentration, specific, small molecules to eliminate off-target effects and improve translational relevance. We have identified several targets of AMPK, previously not described in this area, including leptin and adiponectin, a reduction inten attributed to adipogenic inhibition. Indeed, most literature has identified leptin and adiponectin as upstream regulators of AMPK signalling, and the effects of AMPK itself on their expression is largely unexplored [65–69]. Using short-term transient AMPK activation, we are now able to identify lasting effects of AMPK activation, without directly inhibiting lipogenesis through ACC phosphorylation, and invoking only modest reductions in adipogenesis. As leptin plays a central role in the regulation of feeding and energy balance, it will be of utmost importance to examine the potential effects of leptin suppression by AMPK *in vivo*. Though leptin deficiency drives obesity in *ob/ob* mice through hyperphagia [70], it is more often leptin resistance through chronic exposure to high circulating leptin, derived from adipose tissue, that promotes pathology in human. It may therefore be beneficial to suppress endogenous leptin under some circumstances. Other studies have shown elevated leptin derived from the gut in response to pathogens, and suppression of leptin signalling has ameliorated the inflammatory response [71,72]. It will be of interest to us and the wider field to examine the relationship between AMPK and leptin in other cell types, and in whole tissue. As the methylation of the leptin promoter is a well-established mechanism of regulation in both cells and *in vivo*, future work will focus on the role of AMPK in long-term epigenetic modification, including alterations in DNA methylation, acetylation, transcription factor binding and adipogenic gene expression. Most importantly, we will focus on the novel regulation of leptin by AMPK, and the possible therapeutic relevance of short-term, cell-specific AMPK modulation in metabolic disease.

There are several limitations to this study that we aim to address in future publications.

All mice used in this study were male, and therefore any sexual dimorphism present in these observations has not yet been acknowledged. Future work will expand into female mice, and into human ADSCs derived from both males and females.

Secondly, we acknowledge key differences in the regulation of adipogenesis between adipose tissue depots, namely between subcutaneous and visceral depots, in both mice and in humans. Due to the nature of our previously identified phenotype in the AMPK gain-of-function mouse model, we have focused on the subcutaneous adipose tissue in this study. Future work is required to evaluate these observations in visceral adipose tissue-derived stem cells, though the culture of these cells is often difficult due to their intrinsic commitment to the adipogenic lineage.

Finally, we recognise adipose tissue as a three-dimensional, heterogeneous organ, with many cell types, circulating factors and micro-environments converging on key signalling pathways. We acknowledge that these findings may not fully represent the effects of short-term AMPK activation in whole adipose tissue, and further work is required to corroborate these exciting data in 3D-/co-culture systems and *in vivo*.

## Data Availability

The authors confirm that the data supporting the findings of this study are available within the article and/or its supplementary materials.

## Open Access

Open access for this article was enabled by the participation of Imperial College London in an all-inclusive *Read & Publish* agreement with Portland Press and the Biochemical Society under a transformative agreement with JISC.

## Abbreviations

ADSC: adipose-derived stem cell
AICAR: 5-aminoimidazole-4-carboxamide riboside
AMPK: AMP-activated protein kinase
ANOVA: analysis of variance
ELISA: enzyme-linked immunosorbent assay
FAS: fatty acid synthase
iWAT: inguinal white adipose tissue
mADSC: murine adipose-derived stem cell
PBS: phosphate-buffered saline
SVF: stromal-vascular fraction
WAT: white adipose tissue
