# Supplementary material for: AMP-activated protein kinase activation suppresses leptin expression independently of adipogenesis in primary murine adipocytes
Source: Biochem J. 2024 Feb 23;481(5):345–62. doi: 10.1042/BCJ20240003 (PMC11088909; doi:10.1042/BCJ20240003)
Supplement: Supplementary Material [file BCJ-481-345-s1.pdf]

Supplementary Figure 1

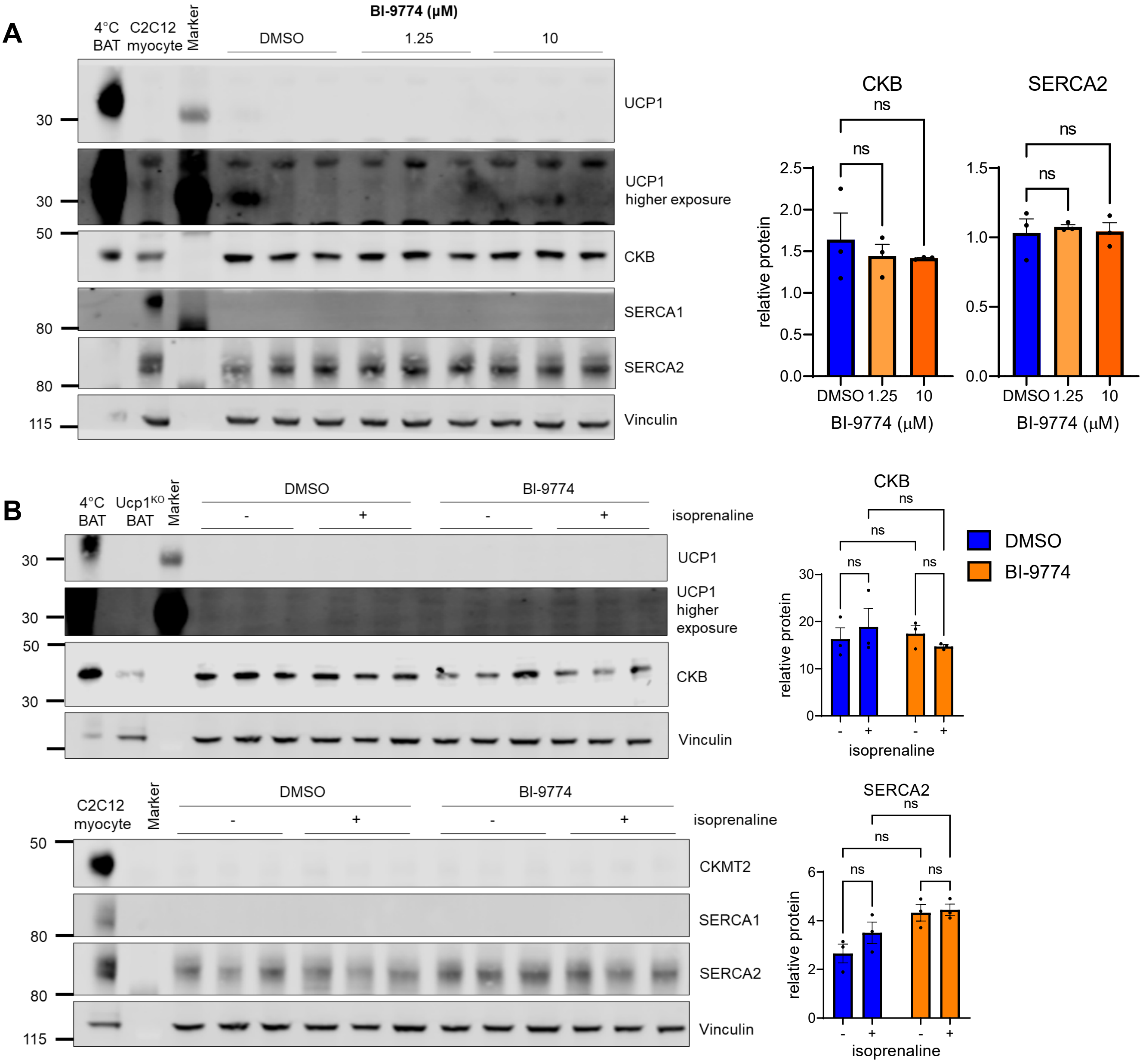

Supplementary figures

Figure S1: AMPK activation by BI-9774 does not promote expression of thermogenic proteins in unstimulated or isoprenaline-stimulated mature adipocytes.

ADSCs were differentiated and after mature adipocyte formation was treated with 1.25 µM and 10 µM BI-9774 for 3 days. **A:** Western blot for thermogenic futile cycle-associated proteins with quantification of CKB and SERCA2 relative to vinculin. **B:** Western blot and quantification of thermogenic futile cycle-associated proteins UCP1, CKB, CKMT2, SERCA1 and SERCA2 in response to 4 h stimulation with 10 µM isoprenaline in mature adipocytes differentiated in the presence/absence of 1.25 µM BI-9774. Data are shown as mean ± SEM (n=3) with statistical significance determined by one-way ANOVA with Dunnett's correction for multiple comparisons. As a control for UCP1 expression, protein lysate from brown adipose tissue (BAT) isolated from either wild-type mice or UCP1 knockout mice maintained at 4°C were compared (upper blot in panel B). For CKMT2 and SERCA1, protein lysate from differentiated C2C12 skeletal muscle myocytes were blotted (lower blot in panel B). CKB: creatine kinase B isoform; CKMT2: creatine kinase mitochondrial 2 isoform; SERCA: sarcoplasmic reticulum calcium ATPase; UCP1: uncoupling protein 1.

Supplementary Figure 2

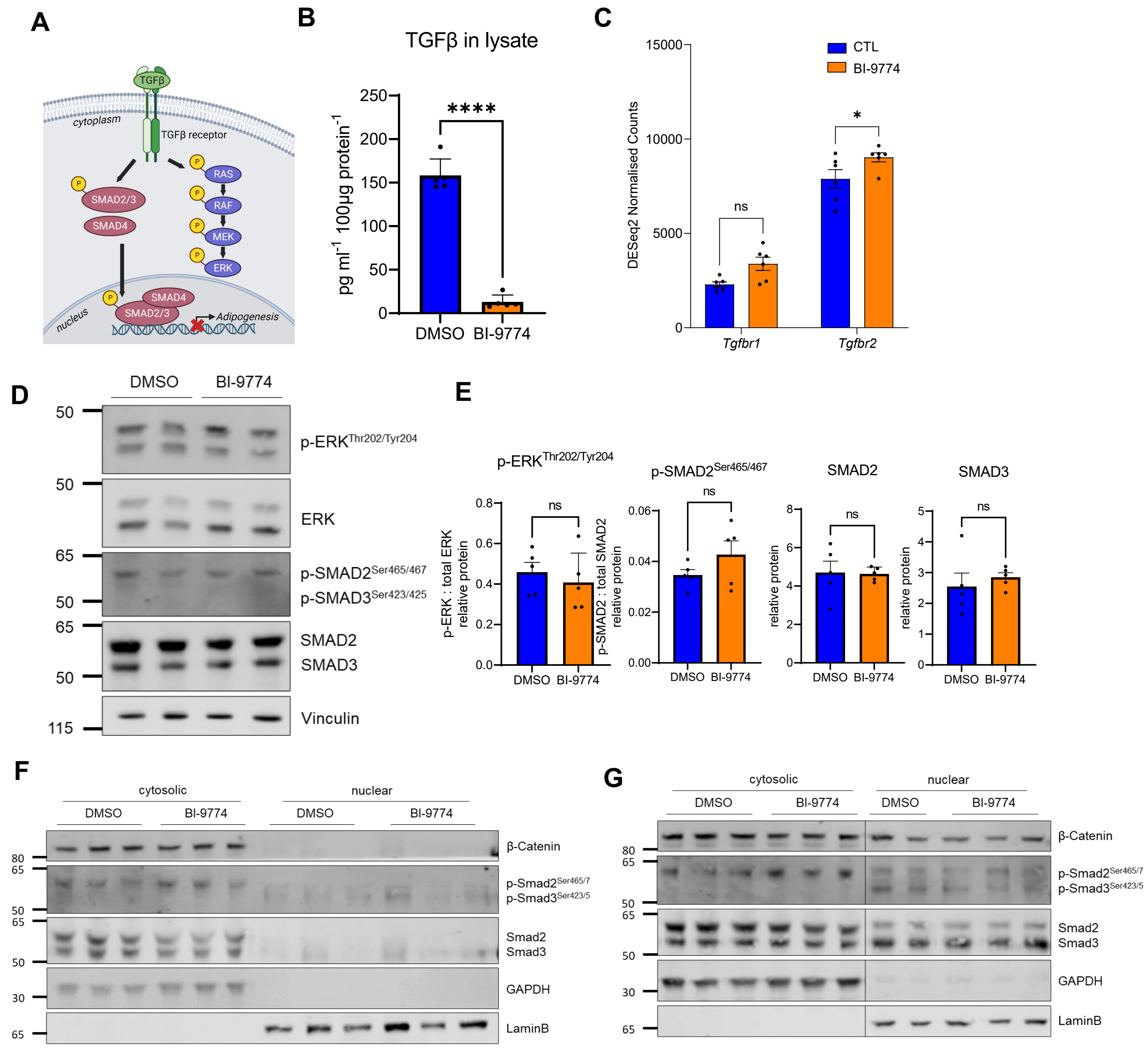

Figure S2: The effects of AMPK on adipogenesis are independent of canonical TGFβ and Wnt signalling

TGFβ downstream targets ERK and SMAD2/3 were assessed at the end of adipocyte differentiation with BI-9774. **A:** Schematic showing principle TGFβ downstream signaling pathways ERK and SMAD (adapted from Principe, Diaz et al. 2017). **B:** TGFβ protein in the cell detected by ELISA. Data are shown as mean ± SEM (n=5) with statistical significance determined by Student's t-test, *p<0.05, ****p<0.0001. **C:** TGFβ receptor (Tgfbr1/2) mRNA expression (DESeq2 normalised counts) generated through RNA sequencing of adipocytes differentiated in the presence or absence of 1.25μM BI-9774. Data are shown as mean ± SEM**,** *p<0.05, with statistical significance determined by two-way ANOVA followed by Šídák's multiple comparisons test (n=6 biological replicates). **D:** Representative Western blot of the downstream targets of TGFβ with **E**: quantification of p-ERK$^{Thr202/Tyr204}$ relative to total ERK, p-SMAD2$^{Ser465/467}$ relative to SMAD2, and total SMAD2 and SMAD3 relative to vinculin. Data are shown as mean ± SEM (n=5) with statistical significance determined by Student's t-test (n=5). **F:** Western blot showing cytosolic and nuclear expression 2 days after adipogenic induction in the presence or absence of BI-9774. **G:** Western blot showing cytosolic and nuclear expression at the end of adipogenesis in the presence or absence of BI-9774 (n=2-3). Abbreviations: ERK, extracellular signal-regulated kinases; MEK, Mitogen-activated protein kinase kinase; RAF rapidly accelerated fibrosarcoma; RAS, Renin-angiotensin-system; SMAD, Suppressor of Mothers against Decapentaplegic; TGFβ, transforming growth factor β.)

Supplementary Figure 3

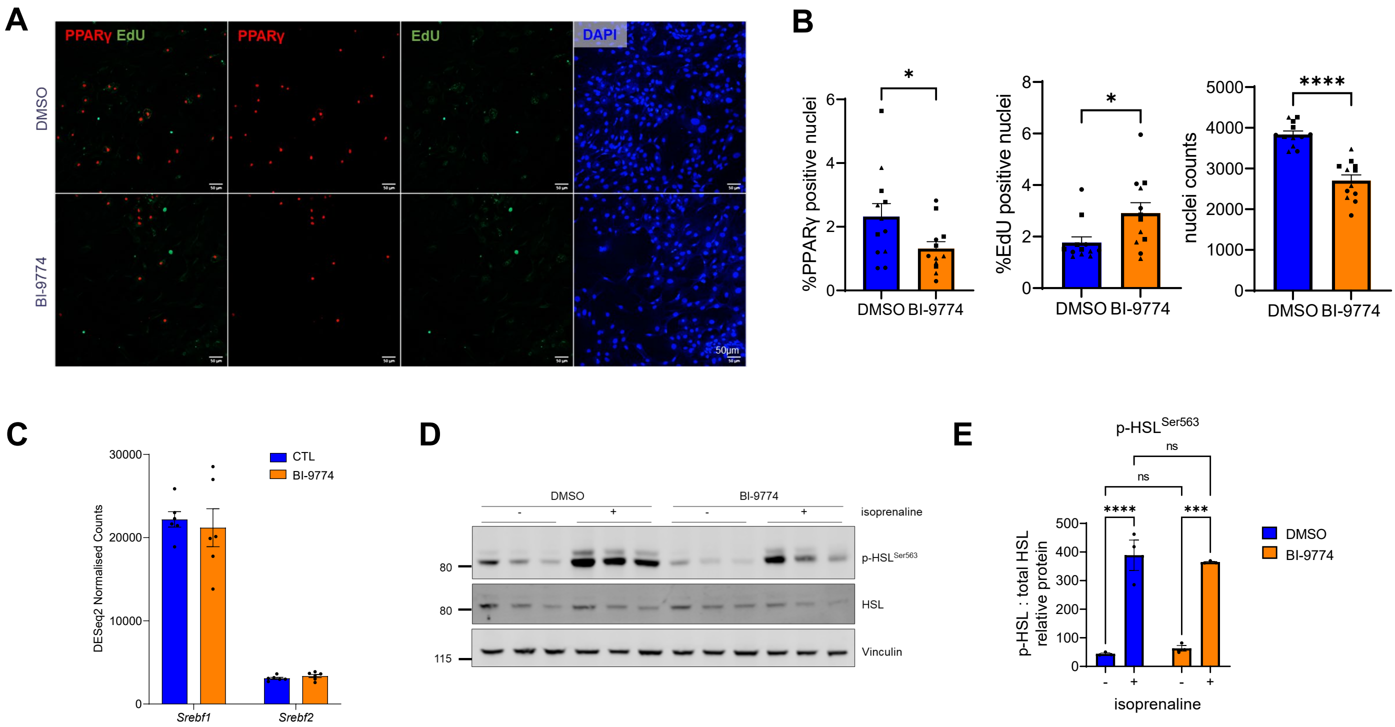

Figure S3: Effects of chronic AMPK activation are not through inhibition of proliferation, SREBP expression or sensitization to isoprenaline-stimulation.

A: Representative images showing non-overlapping PPARγ and EdU expressing nuclei in DMSO and BI-9774 treated cells. **B:** Nuclei counts and percentage of PPARγ and EdU positive nuclei. Data are shown as mean ± SEM (n=3) with biological replicates represented by different shaped symbols and statistical significance determined by Student's t-test with Welch's correction *p<0.05, ****p<0.0001. Scale bar: 50 µm. **C:** Srebp1/2 (*Srebf1/2*) mRNA expression (DESeq2 normalised counts) generated through RNA sequencing of adipocytes differentiated in the presence or absence of 1.25µM BI-9774. (n=6 biological replicates). Western blot **D:** and **E:** quantification of p-HSL^{Ser563} relative to total HSL in basal and isoprenaline-stimulated (10 µM for 4 h) adipocytes differentiated in the presence/absence of 1.25 µM BI-9774. Data are shown as mean ± SEM (n=3), one-way or two-way ANOVA with Dunnett's correction for multiple comparisons, *p<0.05, **p<0.01, ****p<0.0001.
